# Effect of Silane Coupling Agents on Structure and Properties of Carbon Fiber/Silicon Rubber Composites Investigated by Positron Annihilation Spectroscopy

**DOI:** 10.3390/molecules30081658

**Published:** 2025-04-08

**Authors:** Jie Gao, Jiaming Mei, Houhua Xiong, Xiaobing Han

**Affiliations:** Hubei Key Laboratory of Radiation Chemistry and Functional Materials, School of Nuclear Technology and Chemistry & Biology, Hubei University of Science and Technology, Xianning 437100, China; gaojie2019@hbust.edu.cn (J.G.); 13476769851@163.com (J.M.)

**Keywords:** carbon fiber, silane coupling agent, silicon rubber, positron annihilation lifetime spectroscopy, structure–property relationship

## Abstract

The type of silane coupling agent (SCA) has an important influence on carbon fiber (CF) modification efficiency and the properties of the obtained CF-based polymer composites. To quantitatively reveal the effects of SCA type, three kinds of SCA (γ-aminopropyl triethoxylsilane, γ-glycidoxypropyl trimethoxylsilane, and γ-methacryloxy propyl trimethoxylsilane)-modified CF-incorporated silicon rubber (SR) composites were prepared. The microstructure (free volume characteristic and interfacial interaction) of the obtained CF/SR composites was revealed by positron annihilation lifetime spectroscopy (PALS). Based on the results of mechanical, electrical, and thermal properties, the relationship between microstructure and performance was established. This investigation provides a powerful approach to the quantitative description of polymer composite microstructures, which will benefit the construction of structure–property relationships and high-performance polymer composites.

## 1. Introduction

Fiber-incorporated polymer composites have been widely used for weight reduction in vehicles (bikes, automobiles, planes, ships, etc.); this can reduce the emission of carbon dioxide and decrease the consumption of energy [1,2]. The commonly commercialized fibers include carbon fiber [3], glass fiber [4], basalt fiber [5], wollastonite fiber [6], aramid fiber [7], polyethylene fiber [8], and bamboo fiber [9]. Among these fibers, carbon fiber (CF) has attracted attention from many scientists and engineers because of its light weight, excellent thermal, mechanical, electrical properties, and good corrosion resistance [10].

Now, carbon fiber-incorporated polymer composites (CFIPCs) have been widely investigated and applied [11,12]. CF and carbon-black-synergistic-incorporated shape memory polyimide (PI) were developed for the application of electromagnetic shielding (EMI) [13]; the obtained 0.35 mm thick CF/PI film has an average effectiveness of 23.9 dB, with a high transition temperature of 308 °C, which exhibits vast potential in applications for aircraft. MXene nanosheets, Ag nanoparticles, and polydopamine-modified CF have also been developed for the fabrication of epoxy-based EMI materials; the effectiveness of the obtained composites is 45.9 dB, and the flexure strength is as high as 864.6 MPa [14]. To improve the thermal conductivity of CF-based polymer composites, stress-induced orientated CF-incorporated epoxy composites were fabricated [15]. The obtained CF/epoxy composites have a high conductivity of 32.6 W/m·K. To obtain CFIPs with complex geometries, fused deposition modeling/additive manufacturing technology was applied for the fabrication of CF-filled polylactide (PLA) composites [16], and the processing parameters affecting the tensile and flexural strength of the obtained CF/PLA composites were investigated in detail. To enhance the bending resistance of CF/PLA composites, electro-induced continuous CF-incorporated shape memory composites were prepared through four-dimensional printing technology [17]; the recovery rate of shape memory under electric heating was more than 95%, and excellent resistance to different temperatures and bending angles was also observed. The effect of the polymeric matrix (including acrylonitrile butadiene styrene, polybutylene terephthalate, polyamide, and polylactide) on the thermal performance of CF-incorporated composites was also studied [18]. The results revealed that the polymeric matrix has a high impact on the thermal expansion coefficient and thermal conductivity, but it does not affect the specific heat capacity and glass temperature (Tg). The durability of CF/epoxy composites was investigated in water, acidic, and alkaline solutions [19]; according to the results the obtained, composites should avoid contact with acidic environments. In addition, the Arrhenius theory can be used for the prediction of service lifetime under different conditions. To accelerate the sustainable development of CFIP composites, versatile recyclable CF/epoxy composites were developed with imine-containing epoxy hardener [20]; the obtained composite has a high Tg (≥131 °C), solvent resistance, and high tensile strength (≥82 Mpa). Moreover, the obtained composites can be chemically recycled with dissolving or decomposition.

One of the important parameters that affects the properties of CFIPCs is CF surface modification, which can improve the dispersion state of CF and reinforce efficiency [21,22,23]. The widely used surface modifiers for CF include polymer [24,25,26], silane coupling agent (SCA) [27], and metal oxides [28]. Among these modifiers, SCA has attracted much attention for its low cost and high efficiency. The effect of SCA type (vinyl triethoxyl silane, methyltrimethoxyl silane, γ-aminopropyltriethoxyl silane, γ-glycidoxypropyltrimethoxyl silane) on the properties of CF/polyimide composites was investigated [29]; the results demonstrated that the vinyl-end SCA has a significant impact on improving its properties. The influence of SCA chain lengths (methyl, propyl, octyl, dodecyl) on the performance of CF/polyarylacetylene was also studied [30], and the obtained results revealed that the interfacial adhesion increased greatly with the increase of chain length.

The quantitative description of the CFIPC microstructure (free volume fraction and interfacial interaction intensity) is very important for the construction of the structure–performance relationship. The most commonly used quantitative description is interfacial shear strength (IFSS) [29,30], which can correlate the interface interaction with mechanical properties. Polydopamine-modified carbon nanotubes were used for the interface modification of CF/epoxy polymer composites [31]; the various trends of IFSS values share a similar behavior with tensile strength. IFSS has also been used for the microstructure evaluation of polyetherimide-graphene oxide@CF/epoxy [32] and polyethyleneimine-graphene oxide@CF/epoxy [33], which can also explain the change in strength. However, the IFSS cannot correlate the microstructure of CFIPCs with other properties, such as electronic conductivity, thermal conductivity, thermal stability, etc. Due to its decisive advantage in the characterization of microstructure, positron annihilation spectroscopy (PALS) has been applied in the correlation between microstructure and performance [34,35,36,37,38,39]. However, there are few reports about the PALS investigation of CFIPCs.

On the other hand, due to its electrical insulation, superhydrophobicity, temperature resistance, and anti-aging, silicon rubber (SR) is widely used in the industry, including SR-based composites and functional materials [40,41,42]. To improve the mechanical performance of SR materials, montmorillonite (MMT)-incorporated SR was developed [43], and the tensile strength of MMT/SR composites increased from 5.39 to 6.02 MPa. To obtain a durable sensor for health monitoring, highly sensitive graphene/SR composite sensors were fabricated [44], and the obtained sensor exhibited high tunable gauge factors of 27.7–164.5. In addition, CF-incorporated SR has also been prepared for different applications [45,46,47]. However, there is very little in the literature about the effect of CF modification on the properties of the obtained CF/SR composites. Furthermore, there is no quantitative description about the impact of modified CF on microstructure, which goes against the construction of the structure–property relationship.

As mentioned above, different types of SCA (γ-aminopropyl triethoxylsilane, γ-glycidoxypropyl trimethoxylsilane, γ-methacryloxypropyl trimethoxylsilane)-modified CF-incorporated SR polymer composites were fabricated. The free volume fraction and interfacial interaction intensity were quantitatively revealed by PALS, and the correlation between microstructure and properties of the prepared CF/SR composites was established.

## 2. Results and Discussion

### 2.1. Characterization of SCA-Modified CF

#### 2.1.1. SEM Analysis

**Figure 1:**
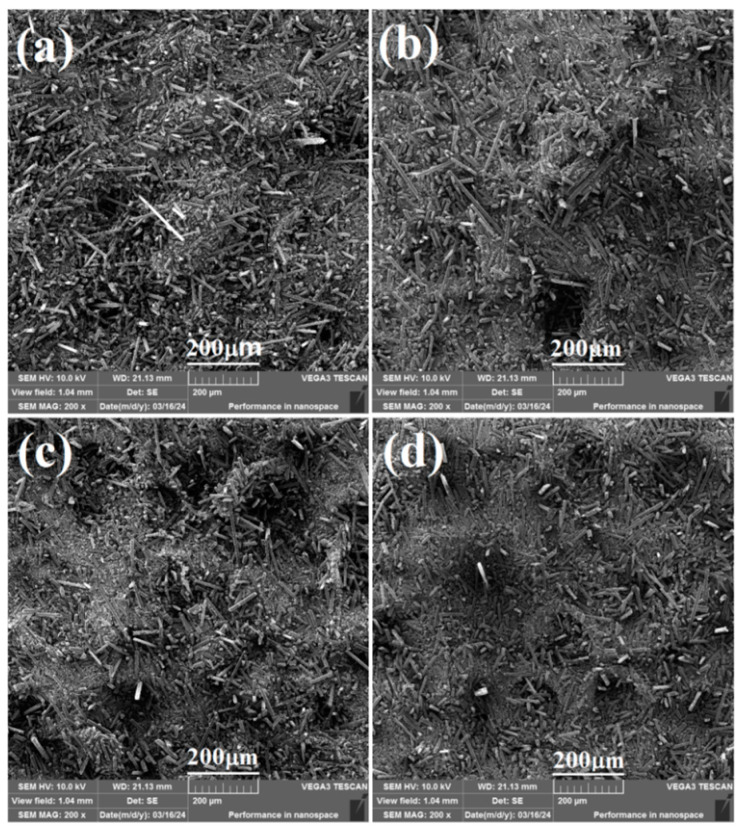
SEM images of (**a**) CF, (**b**) 550CF, (**c**) 560CF, and (**d**) 570CF.

The morphology of CF before and after SCA modification (550CF, 560CF, and 570CF) was revealed by SEM (Figure 1). For the raw CF, the fibers are loose piled, and the surface is clear, and there is no adhesion formed between different CFs. After modification with different SCAs, different degrees of close packing and adhesion were formed between different CFs. This can be ascribed to the intermolecular interactions of the formed polysiloxane coatings, which are obtained with the hydrolysis and condensation polymerization of SCA [25,26,27]. The formed polysiloxane coatings will enhance the dispersion efficiency of CF in SR.

#### 2.1.2. FTIR Analysis

**Figure 2:**
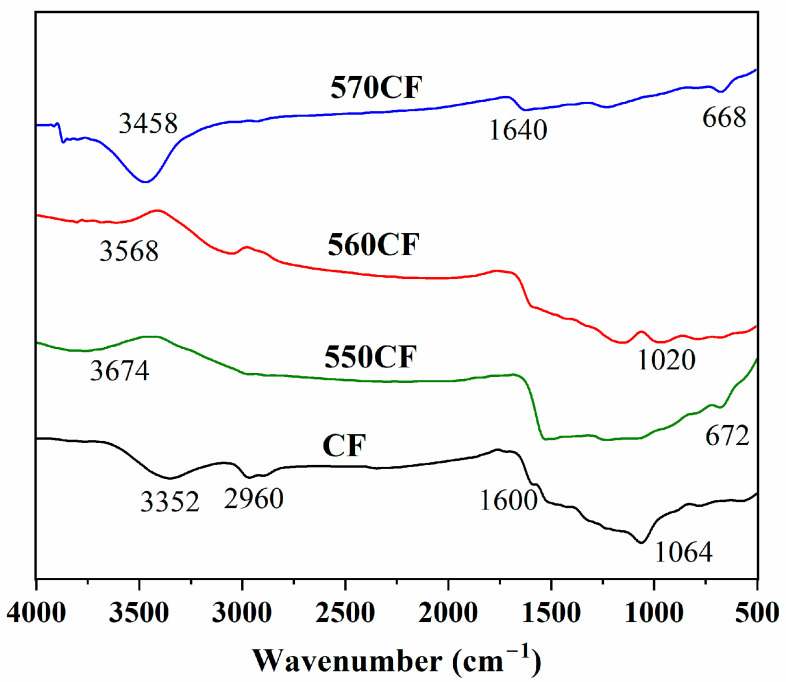
FTIR spectra of raw CF and different SCA-modified CF.

The composition of raw CF and modified CF was revealed by FTIR spectra (Figure 2). For the untreated CF, the broad band around 3352 cm^−1^ belonged to the stretching vibrations of -OH groups. The peaks at 3000~2830 cm^−1^ are associated with the aliphatic groups, and the peak around 1600 cm^−1^ is due to the conjugated structure. The peak at 1064 cm^−1^ corresponds to the stretching vibrations of C-O groups [27,48,49]. After modification with different SCAs, new peaks at 1150~1200 cm^−1^ and 670 cm^−1^ appeared, which were assigned to the vibration of Si-O-C and Si-O-Si, respectively [9,29]. For 550CF, the vibration of N-H bonds is overlapped by broad bands of -OH stretching vibration [9]. For 560CF and 570 CF, the vibration of C-O-C, C=C appeared at 1020 cm^−1^ and 1640 cm^−1^ [29]. All these findings demonstrated the successful modification of CF with different SCAs.

### 2.2. Characterization of CF/SR Composites

To quantitatively investigate the effect of surface modification with different types of SCAs and establish the relationship between microstructure and performance of the obtained CF/SR composites, the free volume fraction (*f_v_*) and interface interaction intensity (β) were revealed by PALS [50,51,52]. The *f_v_* and β are closely related to the mechanical, thermal, and electrical properties of the corresponding composites.

#### 2.2.1. Free Volume Fraction (f_v_) of the CF/SR Composites

According to the relationship between long lifetime τ_3_ and the radius (R) of the free volume hole (Equation (1)), based on the PALS results of τ_3_ and I_3_ of the obtained CF/SR composites, the volume of the free volume hole (*V_f_*) and *f_v_* can be calculated with Equations (2) and (3) [50,51,52].

(1) $$\tau_{3}=\frac{1}{2}{\left[1-\frac{R}{R+\Delta R}+\frac{1}{2\pi }\sin\left(\frac{2\pi R}{R+\Delta R}\right)\right]}^{-1}$$

(2) $$\;V_{f}=\frac{4}{3}\pi R^{3}$$

(3) $$f_{v}=AV_{f}\;I_{3}$$

According to the literature [36,37], the thickness of the surface electron layer ΔR = 1.656 Å, A = 0.0018 Å^−3^, and I_3_ is the concentration of the free volume cavities.

**Figure 3:**
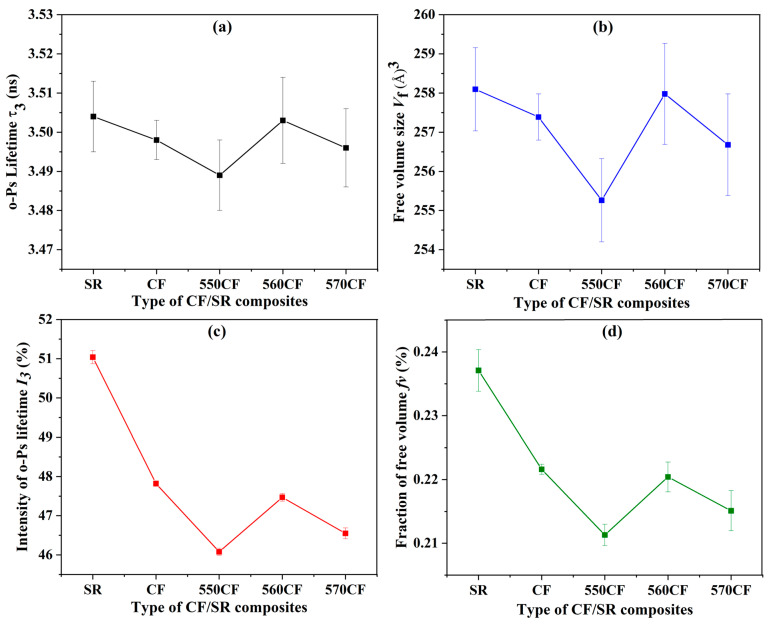
PALS spectra of CF/SR composites: (**a**) long lifetime (τ_3_), (**b**) free volume (*V_f_*), (**c**) long lifetime intensity (I_3_), and (**d**) free volume fraction (*f_v_*).

The τ_3_, *V_f_*, I_3_, and *f_v_* of pure SR and different CF/SR polymer composites are shown in Figure 3. As presented in Figure 3a,b, the τ_3_ decreased with the incorporation of different CFs when compared with pure SR, suggesting the decrease in the average radius of the free volume holes. The lowest τ_3_ and *V_f_* were obtained for the 550CF-incorporated SR composite, which revealed that KH-550-modified CF has the strongest interaction with the SR matrix. The strong interaction can limit the moving of SR chains, leading to the apparent decrease of τ_3_ and *V_f_* [35,36,37].

Compared to pure SR, the I_3_ of all CF-incorporated SR composites decreased dramatically, indicating a decrease in free volume concentration after the incorporation of raw CF and modified CF. This is similar to the reduced graphene oxide-incorporated polycarbonate composites, which can be attributed to the formation of high-density interfacial regions [37,50]. In addition, all modified CF-incorporated SR composites have a lower free volume concentration than raw CF/SR composites, which revealed the good dispersion of modified CF and strong interaction between the modified CF and SR matrix. *f_v_* shows a similar trend as I_3_, and the value of *f_v_* is ordered as follows: SR (0.237%) > CF/SR (0.222%) > 560CF/SR (0.220%) > 570CF/SR (0.215%) > 550CF/SR (0.211%). A lower *f_v_* means stronger interactions and denser composites, which will have an important influence on the properties of the obtained CF/SR polymer composites [50,51,52]. The free volume size (*V_f_*), concentration (I_3_), and fraction (*f_v_*) are determined by both the fiber filling space and the interaction strength between the CF and SR matrix.

#### 2.2.2. Interfacial Interaction Intensity (β) of the CF/SR Composites

The interface interaction between the carbon fiber and polymer chains determines the dispersion condition of CF and the properties of the CF/SR composites; thus, it is of great importance for the precise construction of polymer composites [34,38,39]. PALS not only can reveal the free volume fraction of pure polymers and composites, but it can also probe the interface interaction. The middle lifetime intensity (I_2_) of the composites is always used to calculate the interfacial interaction; the I_2_ of raw CF, pure SR, and all CF/SR composites is listed in Table 1.

**Figure 4:**
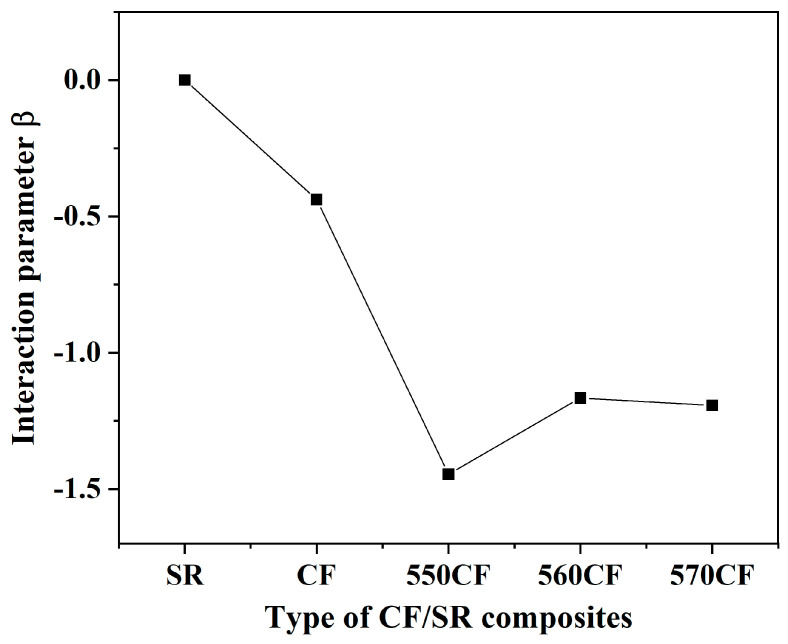
Interfacial interaction intensity (β) of CF/SR composites.

The interface interaction (β) between CF and SR is calculated by the following equation (Equation (4)) [35,49,50]:

(4) $$I_{2}=I_{2}^{CF}W+I_{2}^{SR}\left(1-W\right)+\beta I_{2}^{CF}WI_{2}^{SR}\left(1-W\right)$$

where I_2_ is the middle lifetime intensity of different CF/SR composites, superscripts of CF and SR refer to the carbon fiber and silicone rubber, and W is the weight percentage of CF. The β of different composites is shown in Figure 4, and the absolute values of β in modified CF-incorporated SR composites is higher than that in raw CR/SR composites. This can be attributed to the big difference in surface characteristics between raw CF and the SR matrix, as well as the aggregation of raw CF [35,38]. The absolute values of β for different CF/SR composites are 550CF/SR (1.45) > 570CF/SR (1.20) > 560CF/SR (1.17) > CF/SR (0.44), revealing the best dispersion of KH-550-modified CF and the strongest interaction between 550CF and the SR matrix, which will significantly enhance the properties of the obtained composites.

### 2.3. Properties of Modified CF/SR Composites

#### 2.3.1. Mechanical Properties

**Figure 5:**
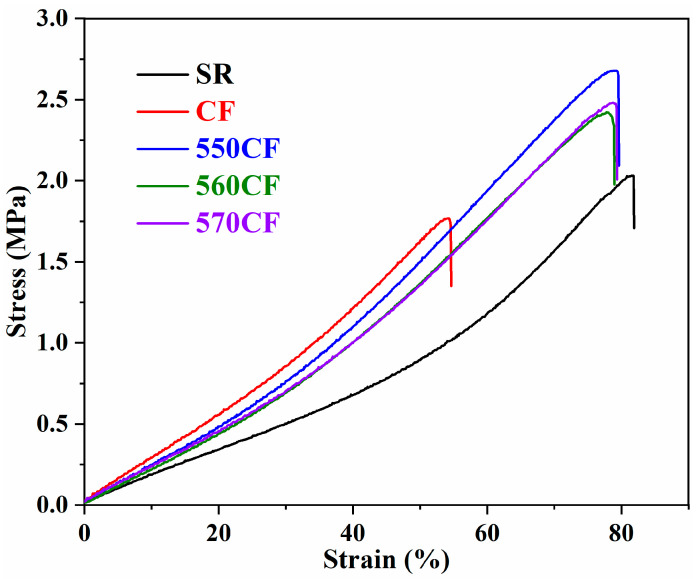
Stress–strain curves of CF/SR composites.

Tensile strength and elongation are important for the mechanical performance of polymer composites, which reveal the highest loading stress and flexibility of the fabricated materials. The stress–strain curves of SR and different SCA-modified CF-filled SR composites are presented in Figure 5.

The tensile strength and elongation at break of all samples are listed in Table 2. Compared with pure SR, only the mechanical performance of raw CF/SR got worse, and the tensile strength and elongation at break decreased by 13.3% and 33.8%, respectively. This can be attributed to the weak interface interaction between raw CF and the SR matrix; the aggregation of raw CF caused stress concentration and deteriorated the mechanical properties [37,48]. The tensile strength of all modified CF-incorporated SR polymer composites increased, and the elongation at break decreased slightly. The optimal mechanical properties were obtained for the 550CF/SR composite, and the tensile strength increased by 32.0%. The mechanical performance of different CF/SR polymer composites is ordered as follows: 550CF/SR > 570CF/SR > 560CF/SR > CF/SR, which is consistent with the variation of interface interaction intensity (β) of the obtained CF/SR composites; a strong interface interaction between SCA-modified CF and a SR polymeric matrix can realize the stress dispersion efficiently [53,54]. The effect of different SCA modifications on tensile strength can also be correlated with their chemical structure; the end-groups of KH-550, KH-560, and KH-570 are amino, epoxy, and vinyl, which have different reactivities toward heat-cured two-component liquid silicone rubber. The liquid silicone consists of Si-H, Si-C=C, and catalyst components, and is cured at high temperature for a long time. For the high-temperature curing with a catalyst, the amino exhibited higher reactivity than vinyl toward the liquid SR, and the epoxy group cannot react; thus, the tensile strength of the obtained composites followed the order mentioned above [4,9].

**Figure 6:**
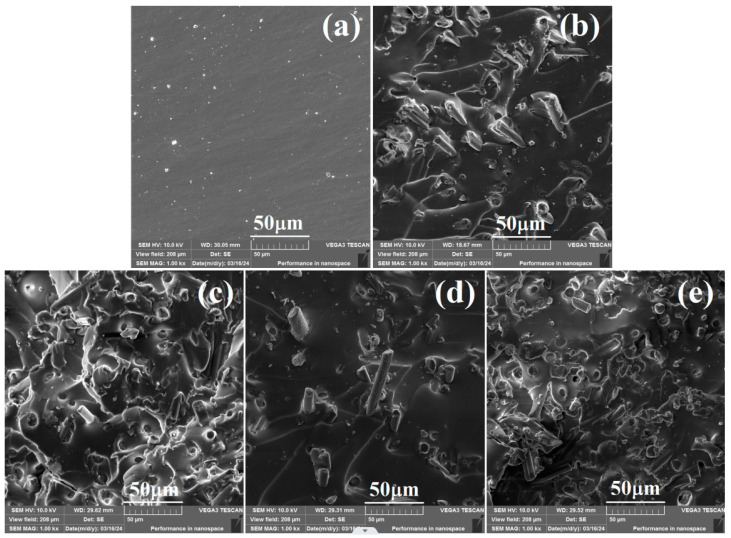
SEM images of the fractured surface of (**a**) pure SR, (**b**) CF/SR, (**c**) 550CF/SR, (**d**) 560CF/SR, and (**e**) 550CF/SR composites.

To demonstrate the dispersion state of different CFs and the interfacial interaction in different CF/SR composites, the fractured surface SEM images of fractured samples in tensile testing are shown in Figure 6. For pure SR (Figure 6a), the fractured surface shows a flat and smooth morphology. With the incorporation of raw and modified CF, the fractured surface became rough, and dispersed CF can be clearly observed. For raw CF-incorporated SR composites (Figure 6b), many raw fibers were pulled out from the SR matrix. What is more, aggregation of raw CF was also observed, which can be attributed to the weak interaction (β) caused by the poor compatible surface [46,55]. For the SCA-modified CF/SR composites (Figure 6c–e), the number of pulled-out fibers almost disappeared except for 560CF/SR. In addition, the dispersion state of modified CF improved obviously, and only a little aggregation of modified fibers was observed within 570CF/SR composites. These results are consistent with the mechanical performance of the obtained CF/SR composites, which can be attributed to the strong interaction (β) formed between modified CF and SR chains [56,57].

#### 2.3.2. Electric Properties

**Figure 7:**
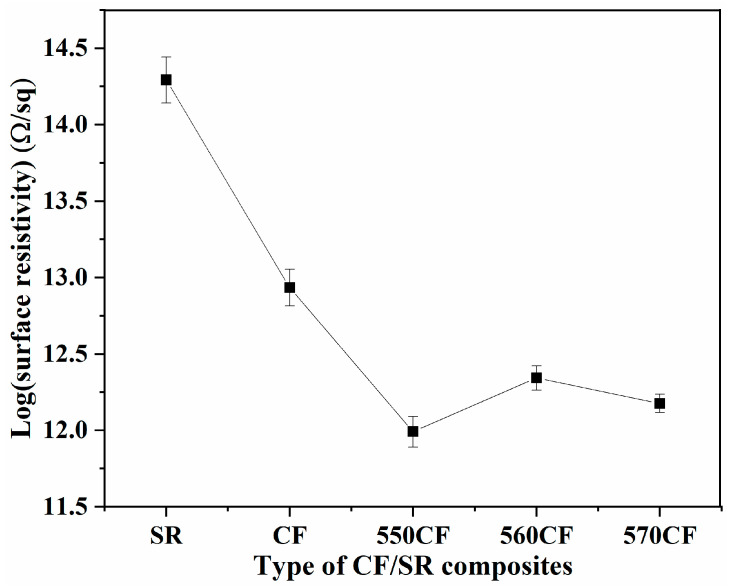
Surface resistivity of SR and different CF/SR polymer composites.

SR-based materials have been widely used in electronic packaging; besides good mechanical performance, the requirement of electronic insulation should also be met [58,59]. The surface resistivity of pure SR and SCA-modified CF-filled SR polymer composites is presented in Figure 7; compared to SR (1.96 × 10^14^ Ω/◻), the surface resistivity of all CF/SR polymer composites decreased due to the introduction of conductive CF [54,55], and the lowest surface resistivity was obtained for 550CF/SR composite (0.98 × 10^12^ Ω/◻). Though the resistivity of all CF/SR composites decreased dramatically, there was still no continuous network constructed because the surface resistivity of all CF/SR materials is higher than 10^12^ Ω/◻; thus, the electrical conductivity was improved through an electron hopping mechanism with non-continuous CF [40,54]. The surface resistivity of different CF/SR composites is ordered as follows: CF/SR > 560CF/SR > 570CF/SR > 550CF/SR, which is consistent with the variation of free volume fraction (*f_v_*). This is due to the low *f_v_* generating a high-density interfacial region, which is beneficial for the hopping electron in the composite [36,50]. The change rule of surface resistivity of the obtained modified CF/SR composites is also related to the chemical structure of different SCAs, and the amino, epoxy, and vinyl end-groups exhibit different electron densities. The nitrogen atom in the amino group contains a lone pair of electrons, and the vinyl group contains two delocalized π electron, which is beneficial for electron hopping. However, there is no free electron within the epoxy group; thus, the surface resistivity of 560CF/SR is higher than that of 570CF/SR and 550CF/SR.

#### 2.3.3. Thermal Properties

For the SR-based polymer composites used as packaging materials, besides electric insulation, they should also possess good thermal performance, such as enhanced thermal conductivity and thermal stability [58,59].

**Figure 8:**
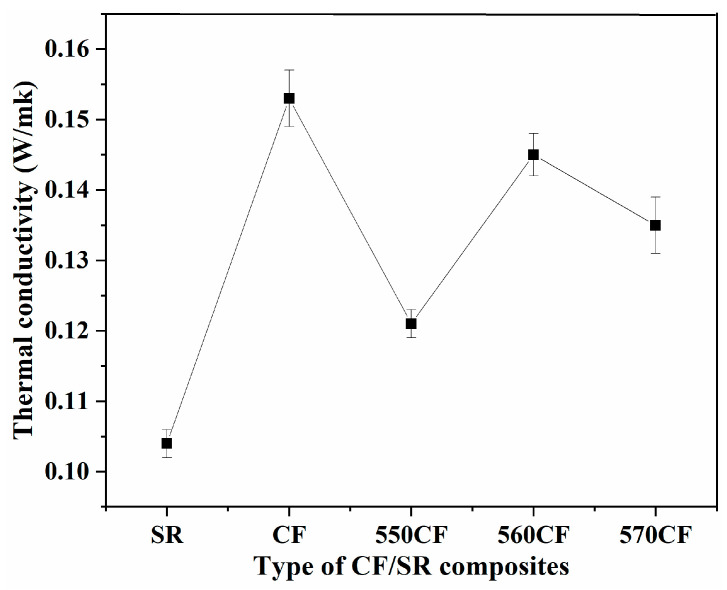
Thermal conductivity of pure SR and different CF/SR polymer composites.

The thermal conductivity of pure SR and all CF/SR polymer composites is presented in Figure 8. SR exhibits a low thermal conductivity of 0.104 W/m·K, which is attributed to the low crystalline degree and severe phonon scattering of the SR matrix. After introducing thermal conductive CF, the thermal conductive performance of all CF/SR composites was improved [40,59]. The highest conductivity (0.153 W/m·K) and the lowest one (0.121 W/m·K) were observed for raw CF/SR and 550CF/SR composites, respectively, which can be attributed to the difference in interface interaction intensity (β). Strong interactions between carbon fibers and SR will induce a high thermal resistance, which can dampen the vibration amplitude and scatter phonons at the interface [50,55]. The strongest β was observed for the 550CF/SR; thus, the highest thermal resistance exists between 550CF and the SR interface, leading to the lowest thermal conductivity of 550CF/SR.

**Figure 9:**
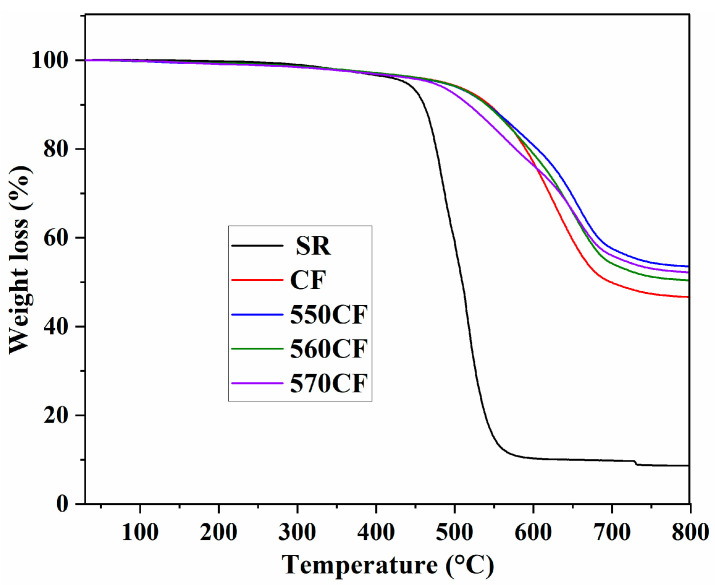
TG curves of pure SR and different CF/SR composites.

The thermal degradation of pure SR and all CF/SR polymer composites is presented in Figure 9. Only 1.0% weight loss was observed for pure SR before 300 °C, demonstrating the good thermal stability of SR. With the increase in temperature, fast decomposition was observed after 430 °C, and only 8.6% residue was obtained at 800 °C [52].

With the incorporation of raw CF and modified CF, the thermal stability of the CF/SR composites significantly improved, which can be attributed to the high thermal stability of inorganic fillers and the barrier effect [52,54]. The temperature at 30% weight loss (T_30%_) and the residue content at 800 °C (R_800_) of all CF/SR composites are listed in Table 3. The highest T_30%_ (648.8 °C) and R_800_ (53.5%) were obtained for the 550CF/SR composite, and the lowest T_30%_ and R_800_ were observed for the CF/SR composite, which is due to the difference of free volume fraction (*f_v_*). A low *f_v_* of the CF/SR composite means difficulty for the diffusion of pyrolyzed molecules and the transfer of heat, as the lowest *f_v_* was observed for the 550CF/SR composite; thus, the highest thermal stability was obtained for the 550CF/SR composite [36,52]. The difference in thermal degradation of the obtained modified CF/SR composites can also be explained with the different chemical structure of SCAs; composites with different cross-linking densities can be prepared with different end-group SCAs. As mentioned above, different SCAs exhibit different reactivities (KH-550 > KH-570 > KH-560) toward the liquid SR [4,9]; thus, the modified CF/SR composite with the highest cross-linking density would be obtained with 550CF, leading to the highest thermal stability.

## 3. Materials and Methods

### 3.1. Materials

The short carbon fibers (CFs, content of carbon ≥ 95%, density 1.75 g/cm^3^) were provided by Toray carbon fiber Co., Ltd. (Guangzhou, China). Concentrated hydrochloric acid was provided by Pingma Group Dongda Chemical Co., Ltd. (Kaifeng, China). γ-aminopropyl triethoxylsilane (KH-550, 99%), γ-glycidoxypropyl trimethoxylsilane (KH-560, 99%), γ-methacryloxypropyl trimethoxylsilane (KH-570, 99%), and ethanol were purchased from HWRK Chemical Co., Ltd. (Beijing, China). Heat-cured two-component liquid silicone rubber (component A 3600 mPa·s, component B 6800 mPa·s, the feeding ration is 1:1 in weight, the optimal curing condition is 150 °C for 3 h) was purchased from Haoming New materials Technology Co., Ltd. (Foshan, China).

### 3.2. Preparation of SCA-Modified CF

**Figure 10:**
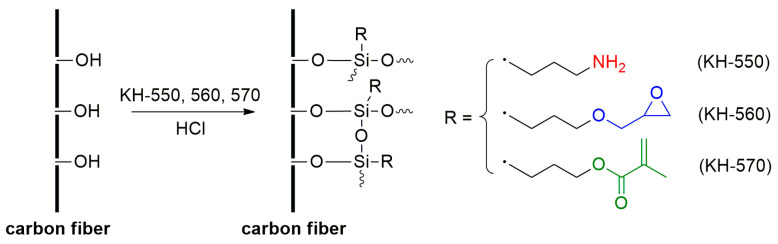
Preparation of SCA-modified CF.

In a typical procedure (Figure 10) [4,9,29], 5 g SCA (KH-550/KH-560/KH-570) was dissolved into 100 mL ethanol, and 5 g CF dispersed into the SCA solution assisted with ultrasound. Then, the pH of the mixture was adjusted to 3 and reacted for 24 h under 60 °C with stirring. Finally, the modified fibers were filtered, washed, and dried to a constant weight. The obtained modified CFs are named 550CF, 560CF, and 570CF.

### 3.3. Preparation of Modified CF-Incorporated SR Composites

Different SCA-modified CF-incorporated SR polymer composites were prepared via a thermal-curing method [40,60,61]. A total of 0.9 g SCA-modified CF was mixed with 3.0 g of component A of liquid SR under 50 °C, assisted with stirring and ultrasound. A total of 3.0 g of component B was added and stirred for an hour. After removing the bubbles from the mixture under vacuum, the sticky liquid mixture was reacted at 150 °C for 3 h within a polytetrafluoroethylene mold. The obtained CF/SR composites were named 550CF/SR, 560CF/SR, and 570CF/SR. For comparison, pure SR and raw CF-incorporated SR polymer composites were also fabricated with the same method, and the prepared samples were named SR and CF/SR.

### 3.4. Characterization of Modified CF and CF/SR Composites

The morphology of modified CF and CF/SR composites was revealed with scanning electron microscopy with SmartSEM 5 (ZEISS Gemini 300, ZEISS, Oberkochen, Germany); the surfaces of all samples were coated with a thin layer of gold, and the acceleration voltage was 20 kV. The chemical structure of modified CF was revealed with Fourier infrared spectra (FTIR), which were recorded by a Avatar 360 Nicolet instrument with OMNIC 9.2 (Thermo Fisher Scientific, Shanghai, China), using KBr pellets with wavenumbers in the range of 4000 to 40 cm^−1^. The tensile strength and elongation were determined using a Shimadzu AG-IC testing machine with TRAPEZIUM 1.5.2 (Shimadzu, Tokyo, Japan), with a strain rate of 50 mm/min. The dumbbell-shaped samples were prepared according to GB/T 528-2009 [62], and the average value was obtained with three pieces of each sample. The electric resistivity of different CF/SR polymer composites was determined by a ST2643 high-resistance tester (Jingge Electronic, Suzhou, China), according to GB/T 31838.2-2019 [63]. The thermal conductivity property of the prepared composites was tested according to GB/T 11205-2009 [64], with a DRE-2C thermal test instrument with DRE Analysis V1.0 (Xiangtan Instrument, Xiangtan, China). The thermogravimeter analysis (TG) of raw CF, modified CF, and different CF/SR composites was determined with a Netzsch TG-209-F3 instrument with Thermal Analysis 4.x (Netzsch, Selb, Germany), with a heating rate of 10 °C/min under an N_2_ atmosphere, in the temperature range of 25 to 800 °C.

### 3.5. PALS Characterization of CF/SR Composites

To quantitatively reveal the microstructure (free volume characteristics and interfacial interaction) of raw CF, pure SR, and the CF/SR composites (CF/SR, 550CF/SR, 560CF/SR, and 570CF/SR), the obtained materials were probed with a PALS technique [34,35,36,37,50,51,52]. The PALS spectrometer was purchased from American Otto Co., Ltd. (Charlotte, NC, USA), and the ORTEC-583 fast-fast coincidence system was constructed with a differential constant ratio timing circuit; the probe consisted of a BaF_2_ crystal and XP2020Q photomultiplier. The PALS was determined with two identical samples sandwiched between a ^22^Na positron source, which was encapsulated in a Kapton foil. For the powder fibers, discs of 2 mm thickness and 15 mm diameter were compressed with a tablet press at 5 MPa for testing. For each kind of sample, a million counts were recorded, and the obtained PALS was resolved by a PATFIT fitting program into three-lifetime components (τ_1_, τ_2_, τ_3_), and their intensities are I_1_, I_2_, and I_3_.

## 4. Conclusions

In summary, the influence of different SCA modifications on the structure and properties of CF/SR composites was quantitatively revealed by PALS. The successful fabrication of different SCA-modified CFs was demonstrated with FTIR and SEM. The SEM images of different CF/SR composites revealed that CF modification with SCA can improve the dispersion and improve the enhanced efficiency. PALS revealed that the *f_v_* of the prepared composites is ordered as follows: SR (0.237%) > CF/SR (0.222%) > 560CF/SR (0.220%) > 570CF/SR (0.215%) > 550CF/SR (0.211%), and the absolute values of β for different CF/SR composites are ordered as 550CF/SR (1.45) > 570CF/SR (1.20) > 560CF/SR (1.17) > CF/SR (0.44). A low *f_v_* is beneficial for the improvement of electrical conductivity and thermal stability, and a high absolute value of β is favorable for tensile strength improvement but unfavorable for thermal conductivity. Therefore, the optimal comprehensive properties were obtained from the KH-550-modified CF-incorporated SR composites, except for thermal conductivity. These findings give deep insight into the influence evaluation of CF modification and the microstructure of the obtained polymer composites.

## Figures and Tables

**Table 1:** I_2_ of raw CF, pure SR, and different CF/SR composites.

Samples	CF	SR	CF/SR	550CF/SR	560CF/SR	570CF/SR
I_2_ (%)	86.21	26.86	33.43	30.79	31.52	31.45
Deviation	1.742	0.159	0.082	0.076	0.084	0.112

**Table 2:** Tensile strength and elongation of raw CF, pure SR, and different CF/SR composites.

Samples	SR	CF/SR	550CF/SR	560CF/SR	570CF/SR
Tensile strength (MPa)	2.03 ± 0.05	1.76 ± 0.04	2.68 ± 0.07	2.36 ± 0.04	2.52 ± 0.05
Elongation (%)	81.4 ± 1.1	53.9 ± 1.5	79.4 ± 0.7	77.3 ± 0.6	78.5 ± 0.4

**Table 3:** Decomposition parameters of pure SR and different CF/SR composites.

Samples	T_30%_ (°C)	R_800_ (%)
SR	486.4	8.6
CF/SR	620.6	46.7
550CF/SR	648.8	53.5
560CF/SR	632.9	50.4
570CF/SR	634.3	52.2

## Data Availability

Data produced in this study can be made available upon a reasonable request.
